# Public awareness of developmental language disorder in Croatia, Italy and Slovenia

**DOI:** 10.1111/1460-6984.12752

**Published:** 2022-06-25

**Authors:** Jelena Kuvač Kraljević, Ana Matić Škorić, Maja Roch, Damjana Kogovšek, Jerneja Novšak Brce

**Affiliations:** ^1^ Department of Speech and Language Pathology Faculty of Education and Rehabilitation Sciences University of Zagreb Zagreb Croatia; ^2^ Department of Developmental Psychology and Socialization University of Padua Padua Italy; ^3^ Department of Special Education and Rehabilitation Faculty of Education University of Ljubljana Ljubljana Slovenia

**Keywords:** developmental language disorder, public awareness, public survey, speech and language pathologist

## Abstract

**Background:**

Previous research, although scarce, has indicated that the general public is still relatively unaware of developmental language disorder (DLD), one of the most common (neuro)developmental disorders. Raising awareness would increase timely involvement in intervention procedures.

**Aims:**

To examine public awareness of DLD in the neighbouring countries of Croatia, Italy and Slovenia, as well as to assess the influence of age, gender and education level on that awareness. Also, to investigate public knowledge about the professionals who recognize DLD and to compare the awareness of DLD with that of other (neuro)developmental disorders in childhood.

**Methods & Procedures:**

A convenience sample of adults living in the countries of the Adriatic region—Croatia (*N* = 92), Italy (*N* = 105) and Slovenia (*N* = 90)—were asked to fill out a paper‐and‐pencil questionnaire (public survey) developed within the Working Group 3 of the COST Action IS1406. Responses were analysed quantitatively as a function of age, gender, education level and country using the *t*‐test and analysis of variance (ANOVA).

**Outcomes & Results:**

Public awareness of DLD is still unsatisfactory in all three countries. Around 70% of respondents reported having heard of DLD; however, only around 20% of Croatian, 40% of Italian and 5% of Slovenian respondents provided an adequate definition of DLD. Differences in research and clinical traditions may explain the observed variations amongst the three countries. Education level was the only variable that was significantly associated with an awareness of DLD in Croatia and Italy: there, more educated people showed a higher awareness and more correct knowledge, which was not found in the Slovenian sample. Respondents generally perceived speech and language pathologists (SLPs) as the professionals responsible for recognizing DLD. Finally, people possess the highest awareness of autism spectrum disorder (ASD), while the awareness of DLD and other (neuro)developmental disorders is equally low.

**Conclusions & Implications:**

Public awareness of DLD varies substantially among the three countries, but there is space for improvement in each of them. The findings of this study build on the existing data from the international group of collaborators, and argue for well‐planned, systematic awareness‐raising activities in the region.

**WHAT THIS PAPER ADDS:**

**What is already known on the subject**

DLD is one of the most common (neuro)developmental disorders, yet it is not well known to the general public. This low awareness hinders timely recognition and adequate intervention, which can have negative psychosocial and emotional consequences for affected individuals. It is known that the awareness of any disorder can depend on one's demographic characteristics, but levels of awareness of DLD are still not examined in detail.

**What this study adds to existing knowledge**

Public awareness of DLD is moderate in Croatia, Italy and Slovenia, but many individuals who report having heard of it appear to misunderstand what it actually is. Of various demographic factors tested, only education significantly influenced public awareness in Croatia and Italy, where more educated people possess greater knowledge. Moreover, respondents generally perceived SLPs as professionals responsible for recognizing DLD.

**What are the potential or actual clinical implications of this work?**

Professionals and researchers should focus on raising DLD awareness in the general public of these three countries, and may need to target different demographic groups accordingly.

## INTRODUCTION

Developmental language disorder (DLD) is one of the most common (neuro)developmental conditions that emerges in early childhood, and it persists throughout the school years and into adulthood. Its prevalence is up to 7.6% in the general population (Norbury & Paul, 2015; Tomblin et al., 1997), and it affects up to 5.8 million children and young people across Europe (COST Action IS1406). Its main characteristics are language skills that are persistently below the level expected for the child, with no identifiable cause such as low general intelligence, neurological injury, hearing impairment or autism (Conti‐Ramsden & Botting, 2008). Since children with DLD at first do not sound different from typical speakers, diagnosis can be delayed, which in turn delays treatment. For this reason, DLD is considered a ‘hidden’ disorder. Cohen et al. (1998) reported that up to 40% of adolescents referred to psychiatric clinics actually had an unsuspected language disorder. As people with DLD grow up, they find their surroundings challenging, and social, emotional and behavioural difficulties emerge. DLD is a lifelong condition, but its behavioural manifestations can be reduced, which can substantially improve outcomes and prevent psychological consequences (Conti‐Ramsden & Botting, 2008; Snowling et al., 2006).

The complexity of DLD has led to longstanding discussions amongst researchers and clinicians regarding terminology, diagnostic criteria and ideal interventions in various settings (Bishop, 2010; Bishop et al., 2016, 2017; McGregor, 2020; McGregor et al., 2020). Differences in the practices of professionals from different sectors in different countries have led to substantial variation in how individuals with DLD are diagnosed and treated. The CATALISE study has synthesized these problems and, based on a complex consensus‐building method in which a multidisciplinary team of 59 experts from English‐speaking countries took part, proposed solutions for them (Bishop et al., 2016, 2017). For example, the team recommended adopting the term DLD over specific language impairment (SLI) and to reconsider traditional exclusion criteria in diagnostics.

After establishing the fundamental notions of DLD, some other more practical issues should be addressed. This includes the availability of therapy and timely involvement in intervention procedures, which largely depend on the level of public awareness. Therefore, the first step in ensuring equal access to services and educational opportunities for all children with DLD is to increase general awareness, and to reconsider and renew outdated education and healthcare policies (McGregor, 2020).

In line with the increasing efforts of researchers worldwide who were motivated to start the change, the European researcher community also gathered around a mutual goal. This resulted in a far‐reaching COST Action IS1406 network, ‘Enhancing Children's Oral Language Skills across Europe and Beyond: A Collaboration Focusing on Interventions for Children with Difficulties Learning their First Language’ (principal investigator: James Law). In this action, researchers from 38 European and several neighbouring countries worked on mapping interventions and service delivery contexts across countries, and on building a systematic knowledge base about DLD and the effectiveness of interventions for these children. As a part of the COST Action, this study also aims to broaden insights on public awareness of DLD.

### Public awareness of DLD

Unfortunately, the topic of public awareness of DLD is largely underrepresented. In fact, if one decides to search the literature, he or she would come across only two papers published recently, both based on the survey developed by the Working Group 3 (WG3) within the COST Action IS1406. The same survey is used in the current study, as the idea was to build on the existing findings and to contribute to the accumulation of data on public awareness, initiated first by the leaders and members of the WG3. In their study, Zegan et al. (2019) focused on the awareness of DLD in the Romanian population, and their results showed that more than half of the respondents knew about the term DLD and language therapy. In a much more comprehensive study with regards to the number of languages and countries involved (18 European countries), Thordardottir et al. (2021) showed that people are generally less aware of DLD than they are of other developmental disorders, for example, autism spectrum disorder (ASD). Additionally, the awareness of DLD seems to be lower in most Eastern European countries in comparison with North‐Western Europe. Potential reasons behind a relatively low public sensitivity to DLD could be found in the vagueness of the concept of ‘language’ for the general public and for policymakers (Kamhi, 2004). The public prefers terms that are easy to understand, remember and use in communication with others, and language is not such a term. A preference for less abstract concepts may also help explain why the public is more aware of other (neuro)developmental disorders in childhood, such as stuttering, ASD and attention deficit hyperactivity disorder (ADHD).

These two studies, as well as many previous studies that were focused on the awareness of different communication disorders (e.g., stuttering or aphasia), confirmed that certain demographic factors play a significant role in this respect. Still, the findings are not always consistent. For example, female respondents in general (Thordardottir et al., 2021) and females with higher levels of education are more aware of communication disorders (e.g., Code et al., 2016; Mahmoud et al., 2014). On the contrary, Zegan et al. (2019) did not find the relationship between respondents’ gender and DLD awareness. The influence of age on awareness is even less clear. Thordardottir et al. (2021) found that the youngest respondents have lower than expected knowledge about DLD, whereas in a large international study on aphasia, Code et al. (2016) revealed that younger persons were more aware of this disorder than older people. Additionally, other researchers found no significant association between age and awareness of stuttering (Van Borsel et al., 1999), or of general delays in language development (Mostafa & Ahmed, 2018). Thordardottir et al. (2021) pointed out that from several background factors, education level has the strongest influence on DLD awareness, followed by age and income.

Besides the general knowledge of DLD in terms of its emergence, manifestations, consequences and clinical and educational needs of this population, the concept of awareness should also encompass the knowledge about professionals trained to provide adequate therapy and support. Studies in different countries have revealed different levels of public awareness of speech and language pathologists (SLPs) and their work (e.g., Breadner et al., 1987; Mahmoud et al., 2014; Parsons et al., 1983). Even in surveys where respondents claimed to have heard of these professionals, further analysis indicated that they may have been ignorant of what SLPs actually do (Breadner et al., 1987; Mahmoud et al., 2014; Parsons et al., 1983). Zegan et al. (2019) reported that most of their respondents recognize SLPs as the main experts involved in DLD intervention delivery, but find the parents responsible for recognition of language disabilities.

As the two recent studies—Thordardottir et al. (2021) and Zegan et al. (2019)—this study is also a part of the larger work of the COST Action and its WG3 and presents the findings at the level of public awareness of DLD and of professionals responsible for recognizing and treating its signs and symptoms. Concretely, the focus is put on the level of public awareness in three European countries: Croatia, Italy and Slovenia. Of the three countries, only Croatia has been involved in the large European study. Nevertheless, it is included in the current study as well for at least two reasons. First, this study addresses additional issues that were not addressed by Thordardottir et al. (2021), that is, it analyses how the public sees experts involved in prevention and intervention procedures. Second, this study compares the three neighbouring countries to get an idea of the awareness of DLD specifically in this region.

The three countries are referred to below as the countries of the Adriatic region, since they are located on the Adriatic Sea and share a common geographical and historical context. Moreover, they share certain features of culture and language, and largely influence each other in various aspects of SLP education. The education of SLPs stems from 1958 in Slovenia, 1962 in Croatia, and 1968 in Italy (De Cagno et al., 2019; Kuvač Kraljević et al., 2019; Novšak Brce & Kogovšek, 2019). Since the mid‐1980s, SLPs in Croatia have been researching and treating DLD and have been keeping up with global changes in terminology, diagnostic procedures and therapeutic approaches (Kuvač Kraljević et al., 2019; Ljubešić, 1995). In Italy, SLPs have worked with DLD populations since the late 1960s, during which time they have gradually shifted their focus from treating and investigating only language difficulties behind DLD to investigating also the impact that language difficulties have on one's socio‐psychological functioning (De Cagno et al., 2019). In Slovenia, language disorders were first mentioned in Zdravko Omerza's (1959), yet since then there has been no systematic research on DLD in the country. As a result, there is still no clear consensus about the state of DLD research and treatment there (Novšak Brce & Kogovšek, 2019). Although Italy has the shortest educational tradition in comparison with the other two countries, its formalization of SLP education and rapid growth in the number of schools across different universities ensured ongoing research of various disorders, those of language, as well. On the contrary, in Croatia and Slovenia the research has been concentrated exclusively at one university in each country.

The members of the WG3 were the impetus for this work, and the initiative for developing the public survey came from them. The authors of the current paper were also actively involved in the COST Action and wanted to build up on the ideas of WG3 members. By including more countries and by extending the analyses, we can expand the data on public awareness of DLD on the map of Europe and fill the gap in the literature regarding this topic.

### Aims of the current study

The aim of this study is to examine public awareness of DLD in each of the three neighbouring countries of Croatia, Italy and Slovenia with respect to demographic factors. Furthermore, the aim is to investigate public knowledge about professionals who recognize DLD and their acquaintance with other (neuro)developmental disorders, at the level of the Adriatic region.

The specific objectives are as follows:

- To examine the levels of public awareness of DLD with respect to age, gender, education level and country of residence.
- To examine respondents’ knowledge about the professionals responsible for recognizing DLD and providing services to affected individuals.
- To determine public awareness of DLD in comparison with other (neuro)developmental disorders in childhood.

## METHODS

### Participants

Respondents were recruited from the general public in Croatia (*N* = 92), Italy (*N* = 105) and Slovenia (*N* = 90) using a convenience sampling method (Table 1). According to the estimates provided by the Statistical offices of European Union (EU) countries, in 2019 the Republic of Croatia had around 4.076 million inhabitants, Italy around 60.36 million and Slovenia around 2.081 million (ECAS, 2019). Participants came from diverse parts of each country and belonged to a broad age range, falling into the categories of ‘younger’ (18–39 years), ‘middle age’ (40–59 years) and ‘older’ (60+ years). Participants also came from a broad range of education levels, categorized as ‘primary/secondary’ (less than 12 or 13 years of formal education, depending on the country) or ‘higher’ (more than 12 or 13 years of formal education). None of the respondents was related to the study investigators or to the data collectors, nor did any of them report being related to an SLP or being an SLP themself.

**TABLE 1:** Demographic characteristics of the respondents from the three countries

		Country			
Characteristics		Croatia	Italy	Slovenia	Total
Participants (*N*)		92	105		287
*Age distribution*					
Younger	18–39 years	31	49	30	110
Middle age	40–59 years	31	29	30	90
Older	60+ years	30	27	30	87
*Gender distribution*					
Male		42	46	32	120
Female		50	59	58	167
*Education level*					
Primary/secondary		46	52	63	161
Higher		46	53	27	126

### Materials

The public survey on DLD (Thordardottir et al., 2021), originally developed in English by the members of WG3 of COST Action IS1406, was translated and back‐translated by the study investigators in each country to ensure that all three language versions addressed the same issues and had a consistent interpretation of the responses. The terminology and all labels used in the survey were discussed during the WG3 meetings. The public survey consists of five sections asking questions about respondents’ demographic characteristics (items 1–10), characteristics of DLD (11–19), management/interventions for persons with DLD (20–23), the role of parents (24–27) and preferred dissemination activities to increase public awareness (28–30). Questions were of different types: open ended, closed, dichotomous choice and multiple choice. For the English version of the public survey, see the in the additional supporting information to Thordardottir et al. (2021).

### Data collection, coding and analysis

Data collection was coordinated and initiated by the leaders of the WG3, but organized by the members of each participating country. One or two students from the University of Zagreb (Croatia), University of Padua (Italy) and University of Ljubljana (Slovenia) handed out the paper‐and‐pencil survey to interested people in public areas such as shopping centres, cafés and bus stops; or in the respondents’ homes following prior arrangement. Each respondent filled out the survey individually after the student or the principal investigator had given him or her a face‐to‐face introduction to the study aims. All participants gave written consent for participation and were aware of the fact that they would not be reimbursed for participation. The survey took an average of 10–15 min to fill out.

The surveys were analysed within WG3 of the COST Action IS1406, but data collection was supervised by the WG3 Chair at the McGill University, hence the ethics approval for all participating countries was obtained there prior to the start of data collection (McGill University, Institutional Review Board (IRB): Study A10‐B63‐17A, 15 January 2018). Given that the approval included all fundamental ethics principles (autonomy, beneficence, non‐maleficence and justice) and since there were no other cultural and social specificities related to the three countries, the ethics approval was accepted as a valid tool at the three national levels. After data collection, all answers were translated to English to assure mutual understanding and to avoid translation and coding issues. Answers were coded in an Excel spreadsheet using a single set of codes shared by principal investigators in each country.

In line with the research questions, only responses to eight of the 30 questions were analysed: these included questions about demographic characteristics (items 1–3 and 5), awareness of DLD and other (neuro)developmental disorders (11 and 29), accuracy of the respondent's basic knowledge of DLD (14), and knowledge about professionals who recognize DLD and work with affected individuals (22). The remaining 22 questions were not relevant in the context of this study, but some of them were subject to another publication (Matić et al., 2021).

The responses to all but one of these questions were coded as 1 (‘yes’), 2 (‘no’) or 3 (‘don't know’). Question 14 asked respondents to describe DLD in their own words, and these open‐ended responses were analysed qualitatively, then coded quantitatively as 1 (‘correct’), 2 (‘inadequate’) or 3 (‘incorrect’) as above, based on how accurate they were judged to be. Two points were given to answers that mentioned at least one clinical marker of DLD, such as developmental language delay, poor vocabulary, or difficulty in comprehension and production. One point was given to answers that referred only to difficulties with speech (e.g., hesitation or pauses during speech) or with pronunciation. Such responses were considered to reflect false understanding of the terms ‘speech’ and ‘language’. No points were given to answers that did not mention language, speech or communication. The responses of 10% of participants were recoded by the second investigator, and interrater reliability was found to be higher than 95%.

Data were reported as frequencies and proportions and analysed using descriptive statistics, *t*‐tests, or analysis of variance (ANOVA) with post‐hoc Scheffé, as appropriate. Bonferroni correction was applied to all analyses involving multiple *t*‐tests. Significance was defined at the 1% level, and test results were reported together with effect size. All analyses were performed using SPSS 23.0 (IBM, Chicago, IL, USA).

## RESULTS

In order to inspect levels of public awareness, our first step was to collect data on how many respondents actually heard of DLD. Most respondents in each country reported having heard of DLD: 70.7% in Croatia, 82.8% in Italy and 71.1% in Slovenia (Table 2). In Croatia, the proportions of respondents who had heard of DLD were higher among middle‐aged women and among those with higher education. Similar results were observed for Italy, except that the younger respondents were more likely to have heard of DLD than middle‐aged or older respondents. In Slovenia, the proportions of respondents who had heard of DLD were higher among younger respondents, among women, and among those with primary/secondary education.

**TABLE 2:** Proportions of respondents who indicated that they had heard of DLD (q. 11), stratified by country of residence, age, gender and education level

		**Country**		
**Question or variable**		**Croatia (*N* = 92)**	**Italy (*N* = 105)**	**Slovenia (*N* = 90)**
Have you ever heard of DLD?	No	0.282	0.086	0.267
	Don't know	0.011	0.086	0.022
	Yes	0.707	0.828	0.711
*Demographic characteristics*		**Croatia (*N* = 65)**	**Italy (*N* = 87)**	**Slovenia (*N* = 64)**
Age	18–39 years	0.308	0.425	0.406
	40–59 years	0.415	0.322	0.391
	60+ years	0.277	0.253	0.203
Gender	Male	0.400	0.437	0.328
	Female	0.600	0.563	0.672
Education level	Primary/secondary	0.400	0.460	0.609
	Higher	0.600	0.540	0.391

*Note*: The top three rows report proportions of the complete samples in each country, whereas the lower rows analyse only the subset of respondents who said ‘Yes’.

Respondents who reported having heard of DLD were asked to provide their own definition of the term, which was scored as 0 if incorrect, 1 if inadequate or 2 if correct (see Methods). Among respondents who reported having heard of DLD, low percentages had an accurate understanding of DLD: 19.7% in Croatia, 42.5% in Italy and 4.7% in Slovenia (Table 3). Most respondents from all three countries revealed incomplete or inaccurate knowledge, most often reflecting confusion between ‘speech’ and ‘language’ (e.g., ‘I think those people have a problem pronouncing certain sounds’) or complete lack of knowledge (e.g., ‘I think these people need a lot of work and effort’; and ‘They have an emotional block’).

**TABLE 3:** Proportions of open‐ended definitions of DLD that were judged to be incorrect, inadequate or correct, stratified by country of residence (q. 14)

	**Croatia (*N* = 61)**	**Italy (*N* = 80)**	**Slovenia (*N* = 64)**
Incorrect	0.098	0.138	0.031
Inadequate	0.705	0.438	0.922
Correct	0.197	0.425	0.047

*Note*: There were missing data from respondents who did not complete the open‐ended question: Croatia = 4 and Italy = 7.

We examined more closely the subset of respondents who claimed to have heard of DLD, but provided incorrect, inadequate or correct definitions, in order to examine potential association between the awareness and country of residence, or other demographic factors. We conducted two tests with a 2 × 3 factorial design (2 genders × 3 countries and 2 education levels × 3 countries) and one test with a 3 × 3 factorial design (3 age groups × 3 countries). Means and standard deviations (SD) of the definition scores (0, 1, 2) per age, gender, education level and country are provided in Table 4.

**TABLE 4:** Means and standard deviations (SD) for scores given to the open‐ended definition of DLD provided by respondents who heard of DLD, stratified by age, gender and education level (q.14)

		**Croatia (*N* = 61)**		**Italy (*N* = 80)**		**Slovenia (*N* = 64)**	
**Demographic factor**		**Mean**	**SD**	**Mean**	**SD**	**Mean**	**SD**
Age	18–39 years	1.05	0.69	1.39	0.68	1.04	0.34
	40–59 years	1.12	0.52	1.27	0.72	1.00	0.28
	60+ years	1.06	0.44	1.00	0.59	0.92	0.28
Gender	Male	1.08	0.65	1.28	0.65	1.05	0.38
	Female	1.08	0.49	1.22	0.72	0.98	0.26
Education level	Primary/secondary	1.00	0.50	1.02	0.66	0.95	0.31
	Higher	1.14	0.59	1.49	0.63	1.08	0.28
	Total	1.08	0.55	1.25	0.68	1.00	0.30

*Note*: There were missing data from respondents who did not complete the open‐ended question: Croatia = 4 and Italy = 7.

The first analysis revealed no main effect of age group [*F*(2, 207) = 1.461; *p* = 0.234], whereas the effect of country was significant [*F*(2, 207) = 3.329; *p* = 0.038; *η* ^2^ = 0.031]. Additional post‐hoc Scheffé’s analysis showed the difference to be significant between Italy and Slovenia (*p* = 0.022), in favour of Italy. Interaction between age and country was not significant [*F*(4, 207) = 0.853; *p* = 0.493].

As for the second analysis, there was no main effect of gender [*F*(1, 210) = 0.305; *p* = 0.581], but again there was an effect of country [*F*(2, 210) = 3.625; *p* = 0.028; *η* ^2^ = 0.033] between Italy and Slovenia (*p* = 0.024; post‐hoc Scheffé), again in favour of Italy. Interaction between gender and country was not significant [*F*(2, 210) = 0.059; *p* = 0.943].

Finally, there was a main effect of education level [*F*(1, 210) = 10.583; *p* = 0.001; *η* ^2^ = 0.039] and country [*F*(2, 210) = 4.261; *p* = 0.015], which indicated that persons with a higher education level have greater and more accurate knowledge about DLD. Additional post‐hoc Scheffé’s tests on the variable country again revealed the difference between Italy and Slovenia (*p* = 0.017), in favour of Italy. Interaction between gender and country was not significant [*F*(2, 210) = 2.506; *p* = 0.084].

Our goal was also to examine respondents’ knowledge about professionals responsible for recognizing DLD and providing services to affected individuals. This analysis was based on responses to question 22. In order to get the initial insight into the state of the art in the whole Adriatic region, the data for this analysis were pooled across the three countries. Most respondents recognized SLPs as professionals involved with DLD and with the affected individuals (Table 5).

**TABLE 5:** Proportions of respondents across the Adriatic region (*N* = 287) who identified the indicated professions as being responsible for recognizing, managing and providing support to individuals with DLD

**Question/profession**	**Who has specific knowledge?**	**To whom could a parent turn for help?**	**Who could help a preschool child?**	**Who could help a school child?**
SLP	0.56	0.57	0.56	0.57
Psychologist	0.40	0.38	0.34	0.39
Teacher	0.29	0.47	0.24	0.29
Assistant teacher	0.29	0.24	0.23	0.32
Nurse	0.06	0.08	0.05	0.05
Physician	0.34	0.43	0.27	0.22

Next, through several questions, we explored how SLPs compared with other professionals (psychologist, teacher, assistant teacher, nurse and physician) in the respondents’ perceptions (Tables 6, 7, 8, 9).

**TABLE 6:** Significant differences (*p* < 0.01) in the proportions of respondents from the Adriatic region (*N* = 287) answering ‘SLP’ or another profession in response to q. 22a: Who has specific knowledge?

	**SLP–psychologist**	**SLP–teacher**	**SLP–assistant teacher**	**SLP–nurse**	**SLP–physician**
*t*	3.89	6.80	6.80	15.39	5.43
*p*	0.00001	0.00001	0.00001	0.00001	0.00001
*h*	0.3	0.6	0.6	1.2	0.4

*Note*: Values indicate the *t*‐value for the comparison of proportions, together with effect size (Cohen's *h*).

**TABLE 7:** Significant differences (*p* < 0.01) in the proportions of respondents from the Adriatic region (*N* = 287) answering ‘SLP’ or another profession in response to q. 22b: To whom could a parent turn for help?

	SLP–psychologist	SLP–teacher	SLP–assistant teacher	SLP–nurse	SLP–physician
*t*	4.64	n.s.	8.55	14.71	3.39
*p*	0.00001		0.00001	0.00001	0.0008
*h*	0.4		0.7	1.1	0.3

*Note*: Values indicate the *t*‐value for the comparison of proportions, together with effect size (Cohen's *h*). Non‐significant differences are omitted.

**TABLE 8:** Significant differences (*p* < 0.01) in the proportions of respondents from the Adriatic region (*N* = 287) answering ‘SLP’ or another profession in response to q. 22c: Who could help a preschool child?

	**SLP–psychologist**	**SLP–teacher**	**SLP–assistant teacher**	**SLP–nurse**	**SLP–physician**
*t*	5.43	8.28	8.59	15.94	7.38
*p*	0.00001	0.00001	0.00001	0.00001	0.00001
*h*	0.4	0.7	0.7	1.2	0.6

*Note*: Values indicate the *t*‐value for the comparison of proportions, together with effect size (Cohen's *h*).

**TABLE 9:** Significant differences (*p* < 0.01) in the proportions of respondents from the Adriatic region (*N* = 287) answering ‘SLP’ or another profession in response to q. 22a: Who could help a school child?

	**SLP–psychologist**	**SLP–teacher**	**SLP–assistant teacher**	**SLP–nurse**	**SLP–physician**
*t*	4.39	7.06	6.23	16.29	9.19
*p*	0.00001	0.00001	0.00001	0.00001	0.00001
*h*	0.4	0.6	0.5	1.3	0.7

*Note*: Values indicate the *t*‐value for the comparison of proportions, together with effect size (Cohen's *h*).

### To whom could a parent turn for help?

Respondents identified SLPs as the leading experts to whom the parents could turn if they had a child with DLD (0.57), as they selected them over most of the other professions provided in the survey, especially over nurses (0.08) (Table 5). Only teachers (0.47) were perceived as equally able to provide initial advisory support to parents of children with DLD (*p* = 0.164) (Table 7).

### Who could help a preschool/school child?

Again, respondents primarily recognized SLPs as responsible for providing services to preschool and school‐age children with DLD (0.56 and 0.57 for preschool and school period in the entire sample). Again, nurses were chosen by the fewest respondents (0.05) (Table 5). Effect sizes were moderate to high in all comparisons (Tables 8 and 9).

**FIGURE 1:**
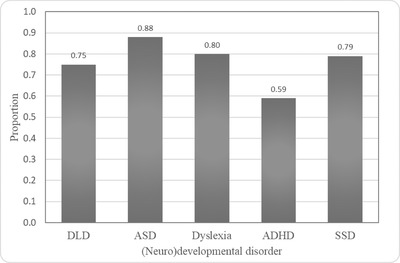
Proportions of respondents in the Adriatic region (*N* = 287) claiming that they were aware of the indicated (neuro)developmental disorder (q. 29). *Note*: ASD, autism spectrum disorder; dyslexia; ADHD, attention deficit hyperactivity disorder; SSD, speech sound disorder.

Lastly, we assessed the level of public awareness of DLD in relation to other (neuro)developmental disorders in childhood across the Adriatic region (Figure 1). A significantly higher proportion of respondents was aware of ASD than of DLD (*t* = –4.07; *p* < 0.00001; *h* = 0.3). On the other hand, contrary to our expectations, participants from all three countries were generally more aware of DLD than they were of ADHD (*t* = 4.14; *p* < 0.00001; *h* = 0.3). The awareness of DLD and dyslexia, as well as of DLD and SSD, seems to be similar in this region.

## DISCUSSION

This study aimed to determine levels of public awareness of DLD in three Southern European neighbours, in which this condition has been relatively understudied: Croatia, Italy and Slovenia. The motivation for this study came from the assumption that higher public awareness can help increase timely involvement in intervention procedures and assure equal educational opportunities for all children with DLD. This study therefore helped broaden the insights into the current level of public understanding of this condition, as well as about the experts to whom the parents and other members of the community could turn in case they recognize signs and symptoms of DLD in their surroundings. Although research on DLD awareness has gradually started to emerge across Europe, there is a lack of findings on individual countries and regions. We aimed to fill this gap by providing the data for the Adriatic region.

Approximately 70% of the respondents in each of the three countries reported that they had heard about DLD. Compared with the data collected at the European level (around 58.9% in 18 countries; Thordardottir et al., 2021), countries from the Adriatic region show higher levels of awareness. According to the more detailed analysis of countries included in the European study, it seems that public awareness of DLD is similar in Croatia, Slovenia, Italy and in countries such as Austria, Lithuania, the Netherlands, Russia and Sweden. These similarities can be explained by historical similarities in DLD research and clinical practice. However, only small proportions of our respondents who reported having heard about DLD actually had accurate knowledge about the disorder: the proportion was 5% in Slovenia, 20% in Croatia and 42% in Italy. The most common misperception that we detected in our samples was confusion between ‘speech’ and ‘language’: respondents often failed to understand that DLD encompasses various forms of communication pathology, not necessarily manifested in speech, including comprehension and expression difficulties in oral and written form. Our results are in line with Kamhi's (2014) claim that the term ‘language’ is insufficiently clear to the public. The term refers to an abstract system much less concrete and therefore less recognizable to the public than ‘speech’, for which our respondents even suggested features such as ‘pronunciation’ and ‘fluency’.

Our analysis suggests that public awareness of DLD in the three countries does not depend significantly on age or gender, but it may depend on education level. This is evident in two out of three countries, Croatia and Italy, where more educated people may be more likely to have heard of DLD and to have accurate knowledge about it. These results are consistent with previous studies that investigated levels of awareness of different disorders of language in populations residing in Europe and across European borders (e.g., Code et al., 2016; Mahmoud et al., 2014), and are also in line with the recent study by Thordardottir et al. (2021) who singled out education level as a demographic factor with the strongest influence on DLD awareness. It seems that prolonged involvement in the higher education system provides access to a variety of social topics, and helps develop sensitivity among the educated people, who then implement it in their surroundings. In contrast to Croatia and Italy, the expected pattern that highly educated people have greater awareness of DLD was not confirmed for Slovenia. A more detailed analysis of the variable *education level* among Slovenian participants showed that 80% of those with ‘lower’ education levels (primary/secondary) obtained a secondary education (e.g., high school). Although general awareness is lowest in Slovenia compared with the other two countries, people with secondary education in Slovenia seem to have access to information about language disorders. This suggests that fine differentiation of the variable education level is important, rather than the dichotomous classification used here. The study by Thordardottir et al. (2021) did not examine differences between countries, but only differences between education levels. If the analyses were conducted between countries, this pattern would potentially occur for 18 countries.

We also found that public awareness of DLD depended significantly on the country: the proportion of respondents aware of DLD was significantly higher in Italy than in Slovenia. This result possibly reflects national differences in research and clinical traditions, especially those related to the number of schools that should not only provide specific knowledge and education to the students, but also promote speech–language therapy, as well as language and other communication disorders in the society. A strong research community certainly ensures visibility of particular phenomena. Although this is not the topic of this study, lack of research for studying DLD in Slovenia is in line with Bishop`s (2010) findings on the close two‐way relationship between research and public awareness.

We further wanted to investigate whether the public from the Adriatic region perceives SLPs as the primary professionals for recognizing and treating DLD. To address this objective on a broader level, we pooled the data from the three countries. An SLP is indeed perceived as the leading expert for these matters. Furthermore, over 50% of all respondents felt that SLPs are the ones parents should turn to for help. Only teachers were placed on a par with SLPs, which shows the respondents’ recognition of teachers as those with a substantial impact on children's language development, since school provides consistent and equitable language input to all children (Norbury et al., 2017). On the other hand, SLPs are correctly perceived as the leading experts responsible for providing services. At least a moderate recognition of the role of SLPs in improving children's language development is one of the initial steps to ensure adequate and timely involvement in speech–language therapy for children who desperately need it. The obtained percentage is higher than the one reported in Zegan et al. (2019), who found that 41% of the Romanian population recognizes SLP as the leading expert.

Certain (neuro)developmental disorders are much more visible or present in the public space than others, and therefore people report increased awareness of their presence. This is especially true for ASD, stuttering and ADHD (Bishop, 2010). Partially in line with these findings, we observed that greater proportions of our respondents were aware of ASD than of DLD (as in Thordardottir et al., 2021). In contrast, respondents were relatively similarly aware of DLD, as they were of dyslexia and SSD. Our results may be explained by the fact that DLD is a ‘hidden’ disorder whose manifestations may not be as obvious as those of ASD. Often, concepts that are more easily perceived spread more quickly through professional and non‐professional communities (Kamhi, 2004). Low awareness of ADHD came as a surprise and is not in line with previous studies that reported significant differences in the awareness of ADHD and DLD in favour of ADHD (58.5–100% awareness in Thordardottir et al., 2021). A possible explanation for the lower percentages obtained in the current study compared with the other studies can be found in the terminology. When asked about awareness of DLD and other (neuro)developmental disorders, three of the four diagnostic labels were given in whole words (*dyslexia*, *autism*, *speech sound disorder*, *language disorder*), whereas only an acronym was given for ADHD. The results might have been different, and respondents might have given more affirmative responses regarding the acquaintance with ADHD, if the key words *hyperactivity* and *attention* had been as clear and as transparent as for the other diagnostic labels provided in the survey. Whatever the reason behind the obtained results, there is obviously room for improvement in terms of awareness of different disorders in the Adriatic region. One solution to this challenge may be long‐term awareness‐raising campaigns (Devilbiss & Lee, 2014), whose features and modalities should be explored in future work.

## CONCLUSIONS

This study has yielded several important findings. First, although the Adriatic region shows higher levels of DLD awareness in comparison with some other parts of Europe, very few respondents actually have adequate knowledge of what it is. Although positive, findings may be misleading if one identifies *hearing about* with *knowing about*. Furthermore, for some of its residents this is especially an ‘invisible’ condition. These are primarily residents with fewer years of education; and residents from Slovenia.

Second, people seem to have gradually developed the recognition of SLPs as the leading experts in providing support to individuals with DLD, and this is crucial for understanding the notions of language, speech and communication.

Finally, our findings still call for more targeted awareness‐raising campaigns that would increase sensitivity of DLD in the general population. Progressive and continuous campaigns on ASD are great examples of how public awareness can be influenced and increased by a focused campaign. Therefore, such examples could be a guide for raising awareness in the field of DLD, and this paper may serve as a baseline of the current state of the art and an indicator of what has to be changed in this part of Europe.

## CONFLICT OF INTEREST

None to declare.

## Data Availability

Research data may be available upon request from the corresponding author.
